# Establishing Reliable Research Data Management by Integrating Measurement Devices Utilizing Intelligent Digital Twins

**DOI:** 10.3390/s23010468

**Published:** 2023-01-01

**Authors:** Joel Lehmann, Stefan Schorz, Alessa Rache, Tim Häußermann, Matthias Rädle, Julian Reichwald

**Affiliations:** Center for Mass Spectrometry and Optical Spectroscopy, Mannheim University of Applied Sciences, Paul-Wittsack-Straße 10, 68163 Mannheim, Germany

**Keywords:** cyber–physical system, sensor data, research data management, FAIR, digital twin, research 4.0, knowledge graph, ontology

## Abstract

One of the main topics within research activities is the management of research data. Large amounts of data acquired by heterogeneous scientific devices, sensor systems, measuring equipment, and experimental setups have to be processed and ideally be managed by Findable, Accessible, Interoperable, and Reusable (FAIR) data management approaches in order to preserve their intrinsic value to researchers throughout the entire data lifecycle. The symbiosis of heterogeneous measuring devices, FAIR principles, and digital twin technologies is considered to be ideally suited to realize the foundation of reliable, sustainable, and open research data management. This paper contributes a novel architectural approach for gathering and managing research data aligned with the FAIR principles. A reference implementation as well as a subsequent proof of concept is given, leveraging the utilization of digital twins to overcome common data management issues at equipment-intense research institutes. To facilitate implementation, a top-level knowledge graph has been developed to convey metadata from research devices along with the produced data. In addition, a reactive digital twin implementation of a specific measurement device was devised to facilitate reconfigurability and minimized design effort.

## 1. Introduction

Initiated through the ongoing digitization, one of the new fields of activity within research concerns the management of research data. New technologies and the related increase in computing power can now generate large amounts of data providing new paths to scientific knowledge [1]. Research is increasingly adopting toolsets and techniques raised by Industrie 4.0 gearing itself up for Research 4.0 [2]. The requirement for reliable Research Data Management (RDM) can be managed by Findable, Accessible, Interoperable, and Reusable (FAIR) data management principles: data must be Findable, Accessible, Interoperable, and Reusable through the entire data lifecycle in order to provide value to researchers [3]. In practice, however, implementation often fails due to the high heterogeneity of hardware and software as well as outdated or decentralized data backup [4,5]. This experience can be confirmed by the work at the *CeMOS* institute of the Mannheim University of Applied Sciences which employs approximately 80 interdisciplinary scientific staff. In the various fields within the institute’s research landscape, such as medical technology, biotechnology, Artificial Intelligence (AI), and digital transformation, a wide variety of hardware and software is required to collect and process the data that are generated, which in initial efforts is posing a significant challenge for achieving holistic data integration.

To cater to the respective disciplines, researchers of the institute develop experimental equipment such as Middle Infrared (MIR) scanners for the rapid detection and imaging of biochemical substances in medical tissue sections, multimodal imaging systems generating hyperspectral images of tissue slices or photometrical measurement devices for detection of particle concentration. Nevertheless, they also use non-customizable equipment such as mass spectrometers, microscopes, and cell imagers for their experiments. These appliances provide great benefits for further development within the respective research disciplines, which is why the data are of immense value and must be brought together accordingly in a reliable RDM system.

Research practice shows that the step into the digital world seems to be associated with obstacles. As an innovative technology, the Digital Twin (DT) can be seen as a secure data source as it mirrors a physical device, also called Physical Twin (PT), into the digital world through a bilateral communication stream [6]. DTs are key actors for the implementation of Industrie 4.0 prospects [7]. Consequently, additional reconfigurability of hardware and software of the digitally imaged devices becomes reality. The data mapped by the DT thus enable the bridge to the digital world and hence to the digital use and management of the data [1]. Depending on the domain and use case, industry and research are creating standardization-independent, new types of DTs. In most cases, only a certain part of the twin’s life cycle is reflected. Only when utilized over the entire life cycle of the physical entity the DT becomes a powerful tool of digitization [8,9]. With the development of semantic modeling, hardware, and communication technology, there are more degrees of freedom to leverage the semantic representation of DTs improving their usability [10]. For the internal interconnection in particular, the referencing of knowledge correlations distinguishes intelligent DTs [11]. The analysis of relevant literature reveals a research gap in the combination of both approaches (RDM and DTs), which the authors intend to address.

In this paper, a centralized solution approach for data processing and storage is chosen, which is in contrast to the decentralized practice in RDM. Typical problems of data management, i.e., many locally, decentrally distributed research data, missing access authorizations, and missing experimental references, which is why the results become unusable over long periods of time, are common issues. The resulting replication of data is followed by inconsistencies and interoperability issues [12]. Furthermore, these circumstances were also determined by empirical surveys at the authors’ institute. Therefore, a holistic infrastructure for data management is introduced, starting with the collection of the measurement series of the physical devices up to the reliable reusability of the data. Relevant requirements for a sustainable RDM leveraged by intelligent DTs are elaborated based on the related work. By enhancing with DT paradigms, the efficiency of a reliable RDM can be further extended. This will form the basis for an architectural concept for reliable data integration into the infrastructure with the DTs of the fully mapped physical devices. Due to the broad spectrum and interdisciplinarity of the institution, myriad data of different origins, forms and quantities are created. The generic concept of DT allows evaluation units to be created agnostically of their specific use cases. Not only do the physical measuring devices and apparatuses benefit in the form of flexible reconfiguration through the possibilities of providing their virtual representation with intelligent functions, but also directly through the great variety of harmonized data structures and interfaces made possible by DTs. The bidirectional communication stream between the twins enables the physical devices to be directly influenced. Accordingly, parameterization of the physical device takes place dynamically using the DT, instead of statically using firmware as is usually the case. In addition, due to the real-time data transmission and the seamless integration of the DT, an immediate and reliable response to outliers is possible. Both data management and DTs as disruptive technology are mutual enablers in terms of their realization [1]. Therefore, the designed infrastructure is based on the interacting functionality of both technologies to leverage their synergies providing sustainable and reliable data management. In order to substantiate the feasibility and practicability, a demo implementation of a measuring device within the realized infrastructure is carried out using a photometrical measuring device developed at the institute. This also forms the basis for the proof of concept and the evaluation of the overall system.

As main contributions, the paper (1) presents a new type of approach for dealing with large amounts of research data according to FAIR principles; (2) identifies the need for the use of DTs to break down barriers for the digital transformation in research institutes in order to arm them for Research 4.0; (3) elaborates a high-level knowledge graph that addresses the pending issues of interoperability and meta-representation of experimental data and associated devices; (4) devises an implementation variant for reactive DTs as a basis for later proactive realizations going beyond DTs as pure, passive state representations; (5) works out a design approach that is highly reconfigurable, using the example of a photometer, which opens up completely new possibilities with less development effort in hardware and software engineering by using the DT rather than the physical device itself.

This paper is organized as follows. At first, Section 2 points out the state of the art and the related work in terms of RDM and DTs. Both subsections derive architectural requirements which serve to evolve an architecture for sustainable and reliable RDM in Section 3. Next, Section 4 outlines specifically picked use cases of the authors’ institute needed for the further implementation in Section 5 as well as the subsequent proof of concept and evaluation in Section 6. Finally, after a discussion that relates the predefined requirements with each other and the implemented infrastructure in Section 7, the work will be concluded and future challenges will be prospected in Section 8.

## 2. Related Work

In order to better situate the present work in the state of the art, the following subsections first show the foundations of RDM, then the developments in the field of DTs. For both focal points, requirements for the development of the later introduced architecture are elaborated, which provides a basis for discussion at the end.

### 2.1. Research Data Management

The motivating force for a reliable RDM should not be the product per se, but rather the necessity to build a body of knowledge enabling the subsequent integration and reuse of data and knowledge by the research community through a reliable RDM [3]. Therefore, the primary objective of RDM is to capture data in order to pave the way for new scientific knowledge in the long term.

To bridge the gap from simple information to actual knowledge generation in order to bring greater value to researchers, data are the fundamental resource that enables the integration of the physical world with the virtual world, and finally, the interaction with each other [13]. The DT as an innovative concept of Industrie 4.0 enables the convergence of the physical world with the virtual world through its definition-given bilateral data exchange. Data from physical reality are seamlessly transferred into virtual reality, allowing developed applications and services to influence the behavior and impact on the physical reality. Data are the underlying structure that enables the DT, thus having a good data management in place provides the realization of the concept [1].

Specifically, in the context of the ongoing advances in innovative technologies, data have evolved from being merely static in nature to being a continuous stream of information [14]. In practice, the data generated in research activities are commonly stored in a decentralized manner on the computers of individual researchers or on local data mediums [5]. A recent study showed that only 12 percent of research data is stored in reliable repositories accessible by others. The far greater part, the so-called “shadow data”, remains in the hands of the researchers resulting in the loss of non-reproducible data sets, devoid of the possibility of extracting further knowledge from this data [4]. In addition, the backed-up data may become inconsistent and lose significance without the entire measurement series being available. According to [15], the most efficient method currently available for transmitting large amounts of data to collaborative partners entails copying the data to a sufficiently large storage drive, which is then sent to the intended recipient. This observation can also be confirmed within the Institute, where this practice of data transfer prevails. Not only is this method inefficient and a barrier to data sharing, but it can also become a security issue when dealing with sensitive data. With such an abundance of data flows, large amounts of data need to be processed and reliably stored, causing RDM to gain momentum within the researcher’s community [14].

Based on the increasing awareness and the initiated ambition towards a reformation of publishing and communication systems in research, the international coalition Force 11 stated the FAIR Principles in 2016. These principles are intended to serve as a guide for those seeking to improve the reusability of their data assets, according to which data are expected to be Findable, Accessible, Interoperable, and Reusable (FAIR) throughout the data lifecycle [3]. The FAIR principles are briefly outlined below within the context of the technical requirements as modeled by the Force 11 Coalition.

1. **Findable:** Data are described with extensive metadata, which are given a globally unique and persistent identifier and are stored in a searchable resource.
2. **Accessible:** Metadata are retrievable by their individual indicators through a standardized protocol, which is publicly free and universally implementable, as well as enabling an authentication procedure. The metadata must remain accessible even if the data are no longer available.
3. **Interoperable:** (Meta)-data utilize a formal, broadly applicable language and follow FAIR principles; moreover, references exist between (meta)-data.
4. **Reusable:** (Meta)-data are characterized by relevant attributes and released on the basis of clear data usage licenses. The origin of the (meta)-data is clearly referenced. In addition, (meta)-data comply with domain-relevant community standards.

While the FAIR Principles define the core foundation for a reliable RDM, there is also a need to ensure that the necessary scientific infrastructure is in place to support RDM [16]. In addition to the FAIR criteria, the concept of a Data Management Plan (DMP) has a significant impact on the success of any RDM effort. The DMP is a comprehensive document that details the management of a research project’s data throughout its entire lifecycle [17]. A standard DMP in fact does not exist, as it must be individually tailored to the requirements of the respective research project. This requires an extensive understanding of the individual research project and an awareness of the complexity and project-specific research data. The actual implementation of a DMP often creates additional work for researchers, such as data preparation or documentation [16]. With the aim of providing researchers with a useful instrument, a number of web-based collaborative tools for creating data management plans have since emerged, such as DMPTool, DMPonline, or Research Data Management Organizer (RDMO) [18].

In addition to the benefits already mentioned, the use of a research data infrastructure facilitates the visibility of scientists’ research as well as identifying new collaboration partners in industry, research, or funding bodies [5,16]. In the meantime, funding bodies in particular have recognized the necessity of effective RDM, making it a prerequisite for the submission of research proposals [16,17].

### 2.2. Digital Twins

The first pioneering principles for twinning systems can be dated back to training and simulation facilities of the National Aeronautics and Space Administration (NASA). In 1970, these facilities gained particular prominence during the 13th mission of the Apollo lunar landing program. Using a full-scale simulation environment of the command and lunar landing capsule, NASA engineers on Earth mirrored the condition of the seriously damaged spacecraft and tested all necessary operations for a successful return of the astronauts. All the possibilities could thus be simulated and validated before the real execution to avoid a fatal outcome of a mishandling [19,20]. The actual paradigm of a virtual representation of physical entities was initiated several years later in 2002. After the first introduction, Michael Grieves further developed his Product Life Cycle (PLC) model, which was later given the term digital twin by NASA engineer John Vickers. The mirrored systems approach was popularized in 2010 when it was incorporated into NASA’s technical road map [21,22,23].

**Figure 1:**
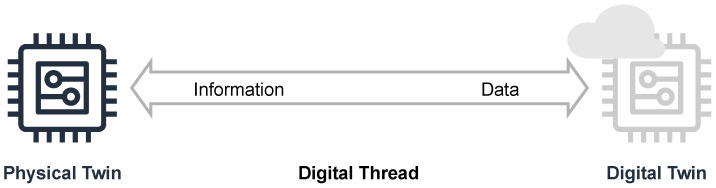
Concept of Digital Twins According to Grieves.

The fundamental concept can be divided into the duality of the physical and virtual world. According to Figure 1, the physical world or physical space contains tangible components, i.e., machines, apparatuses, production assets, measurement devices, or even physical processes, the so-called PTs. In this context, the illustration shows a stylized device of arbitrary complexity on the left-hand side. On the right side, its virtual counterpart is shown in the virtual world or virtual space. The coexistence of both is ensured by the bilateral stream of data and information which is introduced as digital thread. All raw data accumulated from the physical world are sent by the PT to its DT, which aggregates them and provides accessibility. Vice versa, by processing these data, the DT provides the PT with refined information. Each PT is allocated to precisely one DT. One of the goals is to transfer work activities from the physical world to the virtual world so that efficiency and resources are preserved [23]. Especially systems with a multitude of devices require flexible approaches for orchestration. Processes and devices must be able to be varied, rescheduled and reconfigured [24]. Twin technologies as enablers for this provide the greatest possible degree of freedom.

In the evolution of DTs, gradations concerning integration depth can be identified. A distinction is made between digital model, digital shadow, and DT itself. Sepasgozar [25] investigates this coherence and elaborates that digital models are created before the actual physical life cycle of a DT, whereas digital shadows have a unidirectional mirroring of a physical entity. To meet the characteristics of a real twin, communication must be in a bivalent way. Van der Falk et al. [26] deduce DT archetypes from characteristics as well as industry interviews. Starting from *Basic Digital Twins* which, similar to a digital shadow, just represent the state of a physical object, up to increasingly complex twin variations, the following archetypes are further differentiated: *Enriched Digital Twin, Autonomous Control Twin, Enhanced Autonomous Control Twin, Exhaustive Twin.* Starting with the *Autonomous Control Twin*, the DT emerges from its passive role and receives autonomous, intelligent features, which reach their completion in the *Exhaustive Twin*. While the first 3 archetypes can already be found in industry, the more advanced approaches are rather domain-limited or limited to research activities. Grieves [23] also criticizes this and argues that intelligent DTs must shift from their passive role and become active, online, goal-seeking, and anticipatory. DT technologies still need a long time to reveal their full potential. Just by identifying and focusing on the domain-specific challenges this lack of utilizing the opportunities can be tackled [8,27].

Addressing some of these problems, semantic web technologies are inevitably needed labeling the required data streams [28]. Lehmann et al. [29] show that a knowledge-based approach for the representation of DTs is indispensable. Only then interaction between intelligent DTs can take place and they are able to proactively negotiate with others so that, for example, optimal process flows emerge. Sahlab et al. [11] use knowledge graphs to refine intelligent DTs. Particularly in industrial applications, these approaches are distinguished by the management of dynamically emerging DTs. Only through reasoning over the knowledge graphs opportunities for self-adaptation and self-adaptation emerge. Göppert et al. [30] develop a reference architecture for the development of DTs based on an end-to-end workflow addressing definition, modeling and deployment for the description of a pipeline for ontology-based DT creation. Zhang et al. [31] combine DTs, dynamic knowledge bases, and knowledge-based intelligent skills to realize an autonomous framework for manufacturing cells. Due to the manufacturing context, other ontologies are relevant for the definition phase. Therefore, various other requirements and constraints are needed for different applications.

Segovia and Garcia-Alfaro [32] investigate DTs in terms of design, modeling, and implementation and derive functional specifications. Lober et al. [33] also elaborate general specifications for DTs in their work on improving control systems based on them. Introducing a general framework and use case studies, Onaji et al. [34] show important characteristics that DTs must fulfil. In accordance with the insights outlined above and the specifically developed guidelines, the following requirements have to be considered in the present work to realize proper virtual representations of physical entities:

1. **Replication, Representation and Interoperability:** The virtual counterpart of a physical entity should be as detailed as possible, but at the same time as less complex as required without violating the fidelity of the replicated device. A representation should not only include the data of a device but also describe the meaning of this data to lay the foundations for autonomous interoperability.
2. **Interconnectivity, Data Acquisition:** All physical devices must be connected bidirectionally via suitable communication standards. The incoming data must be processed in a time-appropriate manner and reflected in the twin. The data forms to be taken into account can be of a descriptive, static or dynamic nature and must be considered accordingly during processing. Processed information from the DT must also be reflected back into the PT.
3. **Data Storage:** All aggregated data must be stored format agnostically immediately. For reusability, it is necessary to store the data with reference and labeling in suitable storage forms. Not only time but also version, as well as change management, are useful options regarding this.
4. **Synchronization:** Whenever possible, the bivalent data connection should be carried out in real-time and under adequate latency conditions. Both twins should replicate the condition of their counterparts if possible.
5. **Interface and Interaction:** In order to enable collaboration and interaction between and with the twins, suitable interfaces are required. On the one hand, it must be possible for data to be exchanged and accessed by machines, and on the other hand, data must be readable and interpretable by humans providing suitable interaction modes.
6. **Optimization, Analytics, Simulation and Decision-Making:** To gain further advantages, additional features should be accessible through the DTs. Thus, real-time analyses and optimizations as well as independent algorithms for data evaluation can be applied to the data basis of the DT. It should be possible to use AI technologies, establish decision making or use far-reaching simulations, for example. The DT is intended to create context awareness and to facilitate collaborative approaches to reliably choreograph the twins.
7. **Security:** Each entity must comply with the current security standards, i.e., authorization, policies, and encryption. Both privacy and integrity must be preserved. Optionally, the DT could monitor the current security through *what-if* scenarios and initiate countermeasures.

After a detailed examination of both concepts (RDM and DTs), it is obvious that symbiosis of both can draw certain advantages. The requirements for as well reliable as sustainable RDM especially align well with the DT characteristics described above. Both data management and DTs as disruptive technology are mutual enablers in terms of their realization [1]. The analyzed literature reveals a gap in research, which this paper attempts to address. Therefore, the synthesized architecture built out of these pillars is presented subsequently.

## 3. Concept Architecture

**Figure 2:**
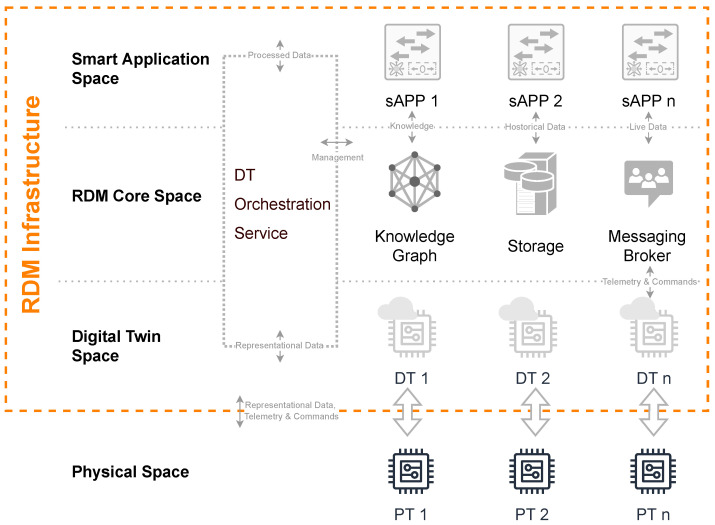
Conceptual RDM Architecture.

After the relevant requirements for a sustainable RDM leveraged by intelligent DTs have been elaborated, the architectural concept will be introduced and aligned regarding these requirements. Subsequently, a decentralized RDM infrastructure is presented facing general data management problems within a research and development institute. Due to the wide range and interdisciplinarity of the institution, countless data of different origins, formats, and quantities are generated. In the authors’ context, data from mass spectroscopy and spectrometry must be specifically assumed, especially in the field of process analytics and medical technology. However, the general infrastructure approaches are to be built up agnostically so that the RDM can also be operated independently of use cases. By enriching it with DT paradigms, the efficiency of a reliable RDM can be further expanded. Thus, not only the physical measuring devices and appliances benefit in the form of flexible reconfiguration facilitated by the possibilities of incorporating their virtual representation with intelligent features, but also directly the wide variety of harmonized data structures and interfaces that are empowered by DTs. Hence, typical data management problems, i.e., many locally, decentrally distributed research data, missing access permissions, and missing experimental references, which is why the results become unusable over long periods of time, are addressed. The proposed approach depicted in Figure 2 tries to overcome these issues.

The overall architecture is split into two main areas: the *Physical Space* in which the physical measuring devices and research equipment are settled and the *RDM Infrastructure* itself which is subdivided into three functional layers: the *Digital Twin Space*, the *RDM Core Space* and the *Smart Application Space*.

From the bottom up there are the physical devices generically referred as *PT 1–PT n*. In accordance with Grieves’ twinning paradigm, these are uniquely linked to their digital counterparts situated within the *Digital Twin Space*. The data-driven representations must be enriched with semantic information content. In this way, it is possible to derive a machine-readable information model. Due to the asynchronous nature of many measurement procedures, the bivalent data pipeline between the two twins is l-driven so that data synchronicity is preserved. The DTs in the *Digital Twin Space* consequently aggregate all data and static, structural information from the *Physical Space* and provide it in a harmonized form to the superimposed infrastructure layers through standard communication interfaces.

The middle layer of the infrastructure, termed *RDM Core Space*, contains the main elements required for reliable as well as sustainable RDM. These include a *Knowledge Graph*, a *Storage* environment, and a *Messaging Broker*. Special attention should be paid to the *DT Orchestration Service* (DTOS), which takes over the choreography of the DTs with all the aggregated data, information, and requests from all participants of the RDM infrastructure that arise.

Starting with the *Knowledge Graph*, it offers itself as an environment for storing all domain-specific knowledge through an ontology. It is intended to organize the entire semantic information of the DT information models. As a sub-discipline of AI, such a knowledge-based approach should bring with it possibilities for reasoning and inferring complex system interrelationships. In this way, DT should be harnessed with intelligence through the *Knowledge Graph*.

The second pillar of the *RDM Core Space* involves examining *Storage* approaches for all accruing forms of data. Because of the different measurement methods and data sources, it also needs different concepts for storage to be considered. For example, some devices deliver a continuous data stream, others asynchronous data points or data sets, and still others preprocessed data, i.e., from imaging measurement procedures. This requires, on the one hand, the necessity of archiving time series data and, on the other hand, a conventional repository-based file system approach. To ensure reusability and interoperability, experiment-specific data must be labeled and versioned. This is also done on a semantic basis so that the experiment data can also be located within the *Knowledge Graph* in order to create intelligent links at later stages and to be able to put data sets into context to other ones.

A *Messaging Broker* is envisaged as a central element that can be accessed anywhere within the architecture. This constitutes an asynchronous, event-driven communications interface for live data of all intended layers and ensures that everyone has non-discriminatory access to all necessary data.

The last major component of the *RDM Core Space* has extra intersections with the lower as well as the superordinate layers. The so-called DTOS manages and orchestrates the entire *RDM Core Space* and thus simultaneously enables intelligent interplay of the DTs. A living part of the DTs is located in the DTOS and gives them functional freedom of action beyond the mostly passive *Digital Twin Space*. Combining the DTs with the stored knowledge within the *Knowledge Graph* results in powerful tools for superposed smart applications. Hence, the responsibility for reading out the DT information models and creating them within the *Knowledge Graph* also lies here. For both data and DTs, lifecycle management is established, so that sustainability and reliability in RDM are created. The DTOS should also provide access to the administration of the DTs as well as the versioning and management of the achieved data in the *Storage*. The DTOS can also be utilized by the *Smart Application Space* through interfaces in order to trigger intelligent functionalities between the DT and the accumulated data.

The *Smart Application Space* is located at the top level of the architecture. At this stage, arbitrary services can consume data via the interfaces of the *Messaging Broker* or the DTOS and use it for their purposes. It would also be conceivable for smart applications to offer their capabilities proactively to the DTs of the devices as a service or even as a service twin. Realizing this, they could also be represented in the *Knowledge Graph* and the *Digital Twin Space* and get into contact with other DTs.

Due to the nature of the research devices, a partly decentralized (data acquisition and preprocessing are commonly facilitated decentrally), mostly asynchronous event-based architecture is needed. Instead of choosing a monolithic software approach, which makes perfect sense on a central system, independent microservices are utilized here. A microservice-based architecture offers the greatest possible advantages in the context of RDM through separate areas of responsibility, independence, autarky, scalability, and fault tolerance through modularity [35]. In order to make the later implementation approaches more comprehensible, a typical use case of the authors’ research institute is presented subsequently.

## 4. Research Landscape and Use Case Description of a Photometrical Measurement Device

After the basic concept architecture for RDM has been presented, the subsequent implementation of a PT will take place on the basis of a specific measuring device and its use cases in order to integrate it prototypically within the RDM architecture as a first application example. Therefore the research and device landscape of the institute will be considered first. The institute conducts interdisciplinary research in the fields of medical biotechnology or medical technology and intelligent sensor technology in order to create synergies between mass spectrometry and optical device development. Based on a variety of covered research areas several devices from different manufacturers as well as self-built ones are used and create a wide-range heterogeneous equipment landscape.

It includes microscopes, cell imagers, various mass spectrometers for generating hyperspectral images, as well as other hyperspectral imagers for specific use cases and optical measuring devices. Some of these imagers and measuring devices were developed, built, and are currently operating at the institute itself. Representatives of these self-developed and manufactured measuring devices are the MIR scanner [36], the Multimodal Imaging System [37], and a multipurpose, multichannel photometer. The MIR scanner is used for generating hyperspectral images of tissue sections and consists of a laser unit with four lasers with different wavenumbers, a detector unit, a focusing unit, an agile mirror unit, and a movable object slide. It is used for frozen section analysis in tumor detection for the identification of tissue morphologies or tumor margins [36]. The Multimodal Imaging System also generates hyperspectral data for tissue sections with various procedures and consists of a modular upright light microscope combined with a Raman Spectrometer, a visible (VIS)/Near-Infrared (NIR) reflectance spectrometer, and a detector unit. Its applications are in brightfield, darkfield, and polarization microscopy of normal mouse brain tissue and an exemplary application provides the ability to the distinction between white and grey matter [37]. The majority of all devices currently do not use a network interface. The resulting measurement data are mostly stored locally and manually collected for analysis purposes. Due to the decentralized processing of the data, no problems arose with regard to security and confidentiality. Likewise, due to the low level of automation, no problems occurred with regard to emerging experimental errors or technical failures. Manual intervention could directly mitigate these errors. In the future, with an increased degree of automation of the RDM infrastructure, issues regarding security, confidentiality but also functional safety have to be taken into account. In addition to the previously mentioned devices, the photometer also serves as an essential component of the institute’s research. For the subsequent implementation of the presented architecture, the feasibility is to be proven on the basis of the photometer.

**Figure 3:**
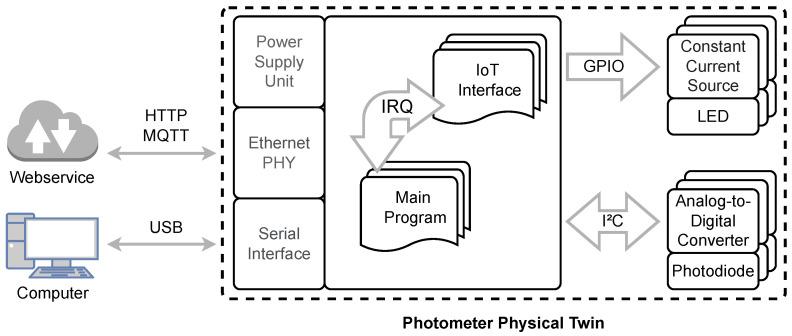
Schematic Layout of the Physical Photometer System.

The developed photometer system schematically shown in Figure 3 essentially consists of three main components. The parts for digitizing the analog sensors, the driver for controlling the light sources, and a powerful microcontroller [38]. These components and their interaction are described in the following. Depending on the application and measuring principle (transmission, reflection), photodiodes with different spectral sensitivities are used. Ideally, these sensors should have high photon sensitivity, fast response time, and low capacitance. Since these criteria are in mutual interaction, an application case-individual consideration is necessary. Exposure of the photodiode causes electrons to be released from the photocathode, resulting in a slight change in the diode’s dark current. A current-to-voltage converter Integrated Circuit (IC) [39] senses this photocurrent from the diode. The Application-Specific Integrated Circuit (ASIC) is a low-noise sensor interface and is suitable for coupling optical sensors with current output. These input currents are quantized into a digital output signal (with up to 16 bits, depending on the integration time). The integration time can be varied between 1 ms and 1024 ms, also the current sensitivity can be varied in steps from 20 fA/*Least Significant Bit* (LSB) to 5000 pA/LSB. Measurements can be continuous or manually triggered. An advantage of the integration of the input signals performed by the device is the resulting significant increase in the dynamic range. Furthermore, high-frequency components are filtered and periodic disturbances with a multiple of the period duration are suppressed.

The microcontroller, connected via an Inter-Integrated Circuit (I²C) interface, is configured in 400 kHz fast mode to communicate with the sensor interfaces. The parameters for writing and reading the Analog-to-Digital-Converter (ADCs) must follow a format specified by the manufacturer. The current state of each ADC (measurement running, measurement finished) can also be queried by reading special registers. Likewise, dedicated General Purpose Input/Output (GPIO) pins can be used to signal the status of the ADCs to the controller as an Interrupt Request (IRQ). This avoids permanent polling of the corresponding register or GPIO pin and saves resources. After a completed measurement, the controller reads the corresponding data packet via the internal interface. Subsequently, a new measurement can be initiated.

In addition to communication with the ADCs, the controller has the task of controlling the LEDs. These light sources are controlled via switchable constant current sources. The selection of the light-emitting diodes used is again very much dependent on the selected measuring principle and the detector. In addition to the wavelength and power of the light source, the rise times ($t_{rise}$) and fall times ($t_{fall}$) in particular must be taken into account in the LED selection. These times can be stored in the controller as parameters of each light source individually. Before starting and after finishing each measurement these specific times are taken into account. Especially for complex measurement setups, low detection limits, and/or short measurement duration, these parameters have a significant influence on the results and the maximum possible scanning speed. Via an integrated USB connection, all parameters can be configured between the photometer and the computer and the raw measurement data can be sent. By means of the built-in ethernet PHY IC DP83825 from Texas Instruments [40], 10/100 MBit communication via ethernet is also possible. An Internet of Things (IoT) interface implemented on the software side, consisting of Hypertext Transfer Protocol (HTTP) server and Message Queueing Telemetry Transport (MQTT) client, enables the connection to further IT infrastructure or web services.

The design of the photometer is highly flexible configurable and modular. According to the selected configuration of the individual components, measurements can be performed in wavelength ranges of ultraviolet (UV), VIS, NIR, and infrared (IR). Furthermore, measurements of e.g., particle sizes can be performed with special probe designs adapted to the task. It is even possible to conduct Raman measurements with probes that are extended by additional optical components. Some specific examples are listed below:

1. **Use of a Scattered Light Sensor for Monitoring the Dispersed Surface in Crystallization.** The specific surface area of the dispersed phase in suspensions, emulsions, bubble columns, and aerosols plays a decisive role in the increment of heat and mass transfer processes. This has a direct effect on the space-time yield in large-scale chemical/process engineering production plants. An easy-to-install optical backscatter sensor outputs the dispersed surface area as a direct primary signal under certain boundary conditions. The sensor works even in highly concentrated suspensions and emulsions, where conventional nephelometry already fails. Trends and limitations found so far for the sensor, which can be used in-line in batch and continuously operated crystallizers, even in harsh production environments and in potentially explosive zones. The specific dispersed surface is directly detected as the primary measurand [41].
2. **Development and Application of Optical Sensors and Measurement Devices for the Detection of Deposits During Reaction Fouling.** In many chemical/pharmaceutical processes, the technically viable efficiencies and throughputs have not been achieved yet because of the reduction in heat transfer (e.g., in heat transfer units, reactors, etc.) due to the formation of wall deposits. Considerable amounts of energy can be saved by reducing or entirely preventing this problematic area. Therefore, a measurement device and its optical and electronic parts were developed for the detection and measurement of deposits in polymerization reactors, simultaneously aiming the in-line monitoring. The design strategy was carried out systematically via theoretical calculations-such as optical ray tracing and photon flux analysis-via test designs, laboratory investigations, and then industrial use. The developed sensors are based on fiber-optic technology and thus can be integrated into the smallest and most complex apparatus, even in explosion-hazardous areas. Critical product and process states in the reactant are detected at an early stage by combining several multi-spectral backscattering technologies. Thus, the formation of deposits can be prevented by changing process parameters [42].
3. **Photometric Inline Monitoring of the Pigment Concentration of Highly Filled Coatings.** Inline monitoring of particle concentration in highly filled dispersions and paint systems using fiber optic backscatter sensors. Due to the miniaturization of the distance between emitter and receiver fiber to <600 µm, the transmitted light can also penetrate high dispersion phase fractions of up to 60%. Due to the measurement setup, both transmission and scattering influences are found in the resulting signal. In this setup, the photometer is configured with detectors and light sources for the red wavelength range (660 nm). The measurement interval of 128 ms is sufficiently small to allow very close monitoring of the measured values [43].

As described above, a number of hardware and software settings and modifications have to be made, especially during the pre-test phase, in order to fulfil the intended task. This usually requires a modification of the firmware of the photometer with the corresponding parameters of the installed components. This time-consuming step, which cannot be performed by every end user, can be eliminated by utilizing the DT of the physical device. Hardware-specific settings can be made comfortably via a Graphical User Interface (GUI). At the same time, a plausibility check of the selected parameters can be realized in a simple way. A misconfiguration of the device can be made more difficult and the end user has the option to check the settings again.

## 5. Implementation

Through the proposed architectural approach on the one hand and the described use case, on the other hand, the prototypical implementation should be outlined on this foundation subsequently. Thus, the general structure and deployment of the main components and their interrelationship will be presented.

**Figure 4:**
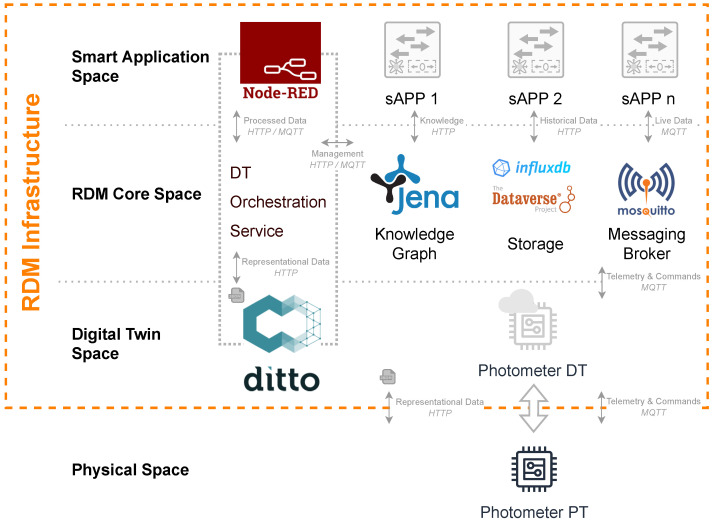
Implementation of the Conceptual RDM Architecture.

Figure 4 illustrates the further developed concept architecture. In each individual layer, the utilized microservice instances are depicted. Instead of just using templates of PTs and their DTs in theory as shown in the concept, the photometer presented in the use case facilitates a complete integration scenario within the RDM infrastructure. It will proceed from the bottom up beginning with the development of the physical photometers representation to further derive its information model for the corresponding DT. Afterward, the entire integration of the *RDM Core Space* is executed by the DTOS. Underlining the implementation and the integration scenario of the photometer a proof of concept will be carried out later.

Every implemented component is built up microservice-based. These microservices are deployed within a distributed server environment at the research institute. Depending on required performance and space, such a microservice infrastructure can be deployed scalable via docker, a docker swarm, or even a kubernetes cluster. Because of the high complexity of such an infrastructure which a reliable and sustainable RDM requires, this section will be further subdivided into several subsections. After prospecting the physical setup of the *Physical Space*, the *Digital Twin Space* will be illuminated. Followed by the *RDM Core Space* in which the interrelations between main objective functionalities of RDM are laid down, a brief overview regarding utilized as well as potentially realizable smart applications within the *Smart Application Space* are shown.

### 5.1. Physical Space

The lower layer of the architecture is the so-called *Physical Space* which contains all physical devices. In this case, the implementation of the *Physical Space* is exemplarily reduced to the photometer introduced in the research landscape. The other pre-presented devices are featured at the proof of concept demonstrating and validating the functionality of the RDM infrastructure. Later, this layer should be extended by the heterogeneous research equipment of the institute.

The photometer previously described above will be used to demonstrate the seamless integration of a research measuring device. For the connection, the IoT interface of the photometer PT is foreseen. The device logs into the Digital Twin Space on every boot sequence via its integrated Representational State Transfer (REST) interface and transmits its structural configuration to it. This auto-deployment ensures that the state between PT and DT is always up to date. All the configurations are embedded in the form of an information model in a JSON file which is directly readable for the overlaying architecture layers. The JavaScript Object Notation (JSON) formatted information model is recognized in Appendix A and exactly mirrors the measuring capabilities and functionalities for the setup of experiments which was outlined before. Important parameters for identification and policy are declared at the beginning of the document. Then the *attribute* part describes the semantic meta contexts and capabilities of the device. Finally, the setting parameters, actuators, and sensors are described as *features*. For reasons of clarity and space, *LED3–LED6*, as well as *adc2–adc4*, have been substituted. Their structure is analogous to the ones shown. Based on the physical structure of the PT, it is one of the main components of the later DT representation. Thus, it serves not only as a data basis for all applications infrastructurally settled above it but also as a semantically enriched information model, which the DTOS uses to describe the device within the *Knowledge Graph*.

### 5.2. Digital Twin Space

At the base of the RDM infrastructure itself, the so-called *Digital Twin Space* contains the DTs of the physical measuring devices. It is based on the open-source project *Eclipse Ditto* [44] which aims to cope with representations of DTs. With a scalable basis, the *Ditto* project offers the possibility of integrating physical devices and their digital representations at a high abstraction level. Not only the organization but also the entire physical-virtual interaction is thus made possible for further back-end applications in a simplified manner. The PT and its DT can be accessed bidirectionally via the provided Application Programming Interface (API). As a result, the *Physical Space* can be influenced by changes within the *Digital Twin Space*. *Eclipse Ditto* is, as the rest of the authors’ infrastructure, built on various microservices. Individual scalability, space-saving deployment, and separation of different task areas as a robust, distributed system, let the project become an universal middleware for the provision of DTs. The essential system components of *Eclipse Ditto* are briefly outlined below.

- **Connectivity Service:** Ensuring friction-less communication between physical devices, their virtual counterparts, and data consuming back end applications the *Connectivity Service* provides a direct interface for various protocols and communication standards such as HTTP, Websockets, MQTT or Advanced Message Queuing Protocol (AMQP). A specially developed, unified, JSON-based *Ditto Protocol* as payload of messages of the listed communication standards opens up numerous interaction possibilities. For example, messages can be also mapped via scripts for preprocessing and after-processing as well as structuring. Furthermore, by using the *Ditto Protocol* the entire *Ditto* instance can be managed, thereby a complete interface is established to interact efficiently with the DTs and their physical counterparts.
- **Things Service:** The *Things Service* contains the actual structure and telemetry representation of the PTs. This abstract representation consists of a simple JSON file. While the first part of the JSON includes the static describing attributes of a DT, such as an unique identifier, the assigned policy, or other e.g., semantically describing properties, the second part contains the dynamic features to which all telemetry data belong. These mirror the constantly changing status of the PTs.
- **Policies Service:** Individual permissions for access and management of the twins, preserving privacy and integrity, are managed by the policy microservice. In order to grant finely graded read and write permissions to certain subjects, *Eclipse Ditto* offers the policy concept that can be easily modified via specific *Ditto Protocol* communication patterns. In addition to extensible certificate-based security mechanisms which *Eclipse Ditto* naively offers, this setup forms the foundation for the fulfilment of modern security standards.

To substantiate the advantages which are brought by *Eclipse Ditto*, the photometer DT should be further instantiated at the *Things Service*. Therefore the before introduced representation form is used and aligned to the requirements of the *Ditto Protocol*. As a result Appendix A with its photometer JSON representation can be reviewed. The part with the key *attributes* at the beginning of the file contains all static and semantic necessary information to draw later benefits from. The second part with the key *features* contains the dynamic telemetry data which are transmitted while operating constantly via MQTT from the PT to the DT and are further consumed by back-end applications or storage purposes. The responsibility for proper connections in direction of the superordinated architectural layers is preserved by the *Connectivity Service*. On top of this middleware-like DT abstraction layer, value-generating features, i.e., the subsequently introduced RDM infrastructure can be constructed.

### 5.3. RDM Core Space

On top of the *Digital Twin Space* the *RDM Core Space* layer is settled. Here, the orchestration of the infrastructure and DTs takes place, the generated data are managed and the communication service is provided. The *RDM Core Space* includes the DTOS, *Apache Jena Fuseki* as *Knowledge Graph*, a combination of *InfluxDB* and *Dataverse* as *Storage* as well as *Eclipse Mosquitto* as *Message Broker*. These instances provide various functionalities that are necessary for the microservices of the RDM infrastructure. Starting on the left with *Apache Jena Fuseki*, the individual infrastructure components are explained in order to subsequently characterize the features of the DTOS and thus fully cover the *RDM Core Space* later on.

**Figure 5:**
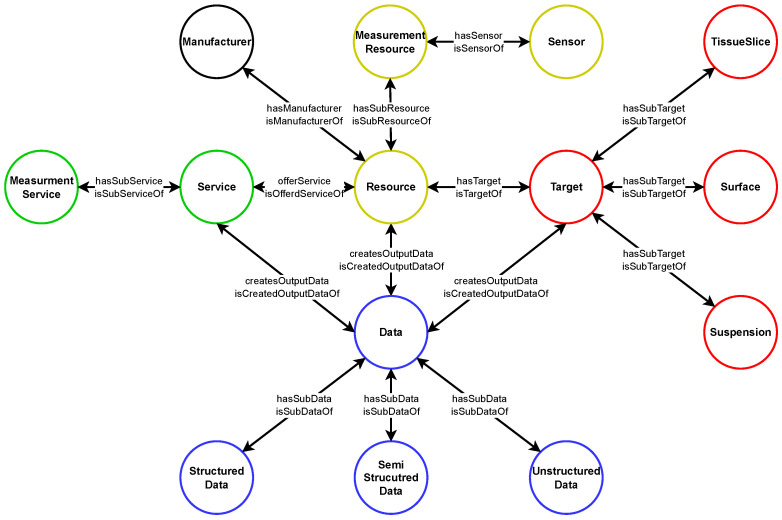
Top-level Knowledge Graph for Research Data Management.

*Apache Jena Fuseki* is a web ontology server that stands out from other alternatives such as Neo4j due to its higher performance. Although Jena Fuseki offers less flexibility in the area of integration of multiple sources, performance is the key criterion for this infrastructure [45]. In comparison to JanusGraph another alternative, Jena Fuseki also predominates in terms of performance [46,47]. In addition, Jena Fuseki uses the common query language SPARQL while Neo4j or JanusGraph use the less common languages Cypher and Gremlin. Based on these mentioned arguments Jena Fuseki is implemented within the infrastructure, however, a more extensive analysis of the suitability of *Apache Jena Fuseki* will be conducted in the future [48]. *Apache Jena Fuseki* is implemented on a dedicated server in a docker container and offers the ability to receive and answer SPARQL queries. It includes a REST API that is used to create the semantic representation of the DTs and to communicate with the microservices across the infrastructure. In addition, a web interface can be used to submit SPARQL queries directly through an input form [49]. In order to process queries, it is necessary that *Apache Jena Fuseki* contains a domain-specific ontology in which the entire semantic information of the DTs information model can be captured. The domain-specific ontology was designed with Protégé according to the requirements of RDM and the DT representation. Figure 5 depicts the top-level ontology for RDM allowing to develop further complex sublevel ontologies in the future due to its modular structure. It enables the DTs to be described semantically with minimal complexity along with contextualizing the generated data of their PTs. The ontology’s basic structure is inspired by Lehmann et al. who presented an ontology for production resources and products [29].

In the context of this work, the ontology was adapted and further fitted to the needs of measurement devices and RDM. At the top hierarchy, the ontology is divided into five logical sections, corresponding to the classes *Resource* (yellow), *Service* (green), *Target* (red), *Data* (blue) and *Manufacturer* (black). Whereby, due to the underlying use case, it has been developed starting from *Resource*. The relationship between the individual classes is as follows. Each *Resource* has a *Manufacturer* and provides a specific *Service* for a particular *Target* and generates specific *Data* from it. These relations can equally be expressed in an inverse manner on the basis of the generated reasoning and inferences as the figure shows. The ontology’s four top-level classes *Resource*, *Service*, *Target*, and *Data* are further divided into sub-classes as shown by the logical sections. The *Resource* (yellow) has the *SubResource Measurement Resource*, which contains the sub-class *Sensor* and enables the *Measurement Resource* to gather data. In order for the *Measurement Resource* to collect data, it must provide a specific *Service*. This Service (green) is provided in the context of the *Services* sub-class as the *Measurement Service*. For a more precise specification of the given device landscape, the top-level class *Target* (red) is divided into the three sub-classes *Tissue Slice*, *Surface* and *Suspension* within the ontology. The classification of the top-level Data (blue) is thereby based on the degree of structurization into Structured, Semi Structured, and Unstructured Data. In the development of this domain-specific ontology, great efforts were made to ensure the best possible foundation for representing the semantic characteristics of the DTs and their data. At the same time, due to its modular structure, it offers future connecting points to roll out the ontology to the entire context of the institute and its requirements. Furthermore, the demonstrated ontology in association with the *Apache Jena Fuseki* web server offers the possibility to provide knowledge-based recommendations via use case specifications, as demonstrated in the proof of concept.

The next essential part of the implementation is the *Storage* which is implemented as a combination of *InfluxDB* and *Dataverse*. The hereby united different storage concepts and cover a maximum number of use cases and fulfil the RDM and DT requirements as effectively as possible. For storing discrete data points *InfluxDB*, an open source time series database is used [50]. The *InfluxDB* stands out from other popular time series databases such as Prometheus, Druid, or OpenTSDB due to its query response time [51]. Compared to Prometheus, *InfluxDB* offers an SQL-like query language, the possibility to manage user rights and in-memory capabilities [52]. In addition, the *InfluxDB* features a better compression ratio than Druid and OpenTSDB. These advantages make *InfluxDB* suitable for storing time series data within the infrastructure [51]. *InfluxDB* is able to sign incoming data with a timestamp and classify it into corresponding buckets which can be assigned to sensors in even more detail with the help of further criteria from the DT. The *InfluxDB* comes with a REST API which allows data to be queried or sent as well as a comprehensive web interface, which enables buckets to be searched and data to be visualized making data findable and accessible. Moreover, the web interface allows the creation of dashboards to enable live monitoring. Besides the discrete data points in the sense of measurement series, other data, such as hyperspectral images, are generated at the institute. *InfluxDB* is not suitable for storing this type of data which is why *Dataverse* is also implemented and serves other storage concepts. It is an open-source web application that allows publishing, storing, citing, and providing research data in associated repositories [53]. Besides *Dataverse*, there are other alternatives such as Zenodo for storing various research data in repositories. *Dataverse* is distinguished from Zenodo by its more advanced authentication options and the superior concept for versioning control [54]. As a result *Dataverse* is implemented within the infrastructure. The repositories in the *Dataverse* are called *Dataverses* and can be subdivided for example by working groups or projects. Within the *Dataverses* so-called *Datasets* can be created, in which data, for example from measurements, can be saved. The *Dataverse Project* offers extensive metadata management and makes it possible to describe the individual *Datasets* more exactly which facilitates interoperability of data. Furthermore, the *Datasets* can be directly linked to a publication with a Digital Object Identifier (DOI), allowing the user to extract corresponding citations directly from the web application. Metadata management within *Dataverse* contextualizes the stored data and provides a high level of reusability for other users [55]. *Dataverse* also provides a REST API to upload or query data which simultaneously can be uploaded and searched with different filters for the metadata in a simplified manner via the web application. Thus requirements of different users are served by it. Analogous to *Apache Jena Fuski InfluxDB* and *Dataverse* are also implemented on a provided server within the distributed environment.

The next element of the implementation in the *RDM Core Space* is the message broker, which is implemented by *Eclipse Mosquitto*. It is a message broker that supports the MQTT communication protocol and enables interaction within the infrastructure and its microservices [56]. At this stage of development, MQTT is used due to its less required deployment resources. In the future, however, this communication interface will be enhanced by AMQP due to the buffering and the larger range of functions, in order to thus be able to serve a wider scope of requirements [57]. In this context, *Eclipse Mosquitto* provides the link between the PTs and DTs, the DTOS and DTs, the DTOS and *Node-RED*, and the DTOS and smart applications in general. By connecting the PTs and the DTs, a bidirectional connection of both units is formed and their interconnectivity is ensured. *Eclipse Mosquitto* provides different security levels for the message traffic, ranging from no security to encryption with Transport Layer Security (TLS) and TLS with a client certificate. The implementation currently provides no encryption for the message traffic, but this will be addressed in the future. The broker is located on the provided server for the infrastructure and operates beside the previously presented elements of the *RDM Core Space*.

**Figure 6:**
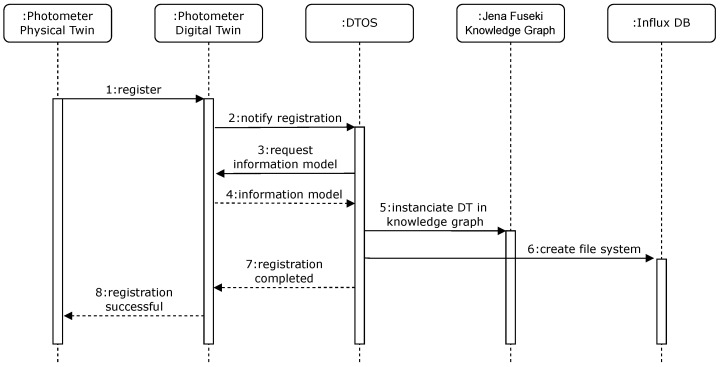
Registration of a Digital Twin within the RDM Core Space.

In addition to the presented instances within the *RDM Core Space*, the DTOS represents a central component at this layer of the infrastructure, which performs different orchestration tasks as an event-driven microservice and is currently implemented as a python-based microservice. It captures the registration of new DTs, creates their semantic representations in *Jena Fuseki* and a file system in the *InfluxDB*. Furthermore, the DTOS connects the *Digital Twin Space* with the *Smart Application Space* and thereby enables intelligent cooperation between the DTs. The sequence chart shown in Figure 6 illustrates the operations inside the infrastructure and the DTOS, which are outlined in more detail below.

The entire process starts with a PT being activated and registering with its underlying information in the DT information model via a HTTP registration request. Through this registration request, a DT is provided for the PT with the help of *Eclipse Ditto*. After the DT is successfully instantiated, it notifies the DTOS of its registration via MQTT. This is the event trigger for the DTOS, which now submits a HTTP request for the DT’s information model. The DT then provides its information model to the DTOS. This information model contains, as shown in the Appendix A exemplarily for the photometer, attributes, and features besides general properties. The general properties and the structure of the JSON with the keys *attributes* and *features* originate from the *Eclipse Ditto* information model and are necessary for the registration as well as the instantiation. All components of the device like sensors or ADCs are included in the key *features*. Additionally, information for the DTOS is included with the variable *regComplete* which is set to false by default. This variable is altered from false to true with the registration of the DT within the infrastructure and controlled by the DTOS to prevent the duplicate creation of the file system in the *InfluxDB* and the semantic information on the *Apache Jena Fuseki* server. The DTOS starts to extract the semantic information about the DT from the information model if the check of the variable *regComplete* results in the fact that the DT has not yet been registered. These semantic information are contained in the key *attributes* and structured as a Resource Description Framework (RDF) triple according to the Web Ontology Language (OWL) to simplify its creation in the *Knowledge Graph*. After the positive check of the *regComplete* variable, the DTOS parses the content of the key *attributes* and generates a SPARQL query using the HTTP POST method and thereby transmits it to *Apache Jena Fuskei* instantiating the DT in the *Knowledge Graph* and all its sensors as new individuals inside the presented ontology. Simultaneously, the DTOS creates a file system for the DT in the *InfluxDB* via HTTP to store its generated data. Subsequently, the DTOS reports the complete registration process to the DT and the DT returns the notification of successful registration to its PT via MQTT. Thus, the PT is fully integrated into the infrastructure and can start generating data that now are saved via the DTOS in the associated file system and made accessible to other microservices as well as the smart applications in the *Smart Application Space*. In addition, the semantic information can now be queried through the *Knowledge Graph* and taken into account in queries about specific device properties.

Besides the orchestration of the DTs, the creation of file systems and semantic representations, the DTOS takes over the lifecycle management for the generated data as well as the DTs. For this purpose, the DTOS monitors the DTs registered in the infrastructure and enables their deregistration if required. During deregistration, the DTOS removes the semantic representations from the *Knowledge Graph* and moves the DT’s data to the long-term archive, which is provided via the *Dataverse* as well as deletes them when they reached the end of their lifecycle. Similarly, the DTOS monitors the generated data over its lifecycle from creation to publication to archiving. In doing so, the DTOS links the data to the associated publications and then moves the data to the archive until it deletes it at the end of the life cycle.

### 5.4. Smart Application Space

Smart applications in the RDM infrastructure are settled at the top of the hierarchy. Applications for the evaluation of experiments and the creation of added value are to be located according to the infrastructure modalities that are as open as possible. Examples of such smart features would be AI algorithms for the evaluation of medical image data, context-based correlation of multi-dimensional parameter fields, optimization procedures for measurement arrangements and much more. Some of the applications already implemented and those planned for the near future are discussed below.

Originally developed by IBM, the open source software *Node-RED* is a tool for flow-based programming and is used for connecting hardware components, APIs, and online services. *Node-RED* provides an editor through the web browser that enables a graphically supported creation of flows with different nodes [58]. With *Node-RED* further microservices for the RDM infrastructure are implemented, such as the knowledge-based recommendation system for measuring devices. With the knowledge-based recommendation system, a tool is implemented in the *Smart Application Space* that facilitates the selection of measuring devices for the end user. For this purpose, an input mask is set up in a *Node-RED* dashboard with which the parameters for a measurement to be performed can be specified. Based on these specifications, the microservice generates a SPARQL query and sends it to *Apache Jena Fuseki*. The response from the *Knowledge Graph* is output in tabular form with the required parameters. With the help of the input mask the required service, the target, and the output data can be defined. In addition, the required wavelength can be specified either as a specific value or as a range. All entries are optional and serve the refinement of the search filter. This recommendation system is featured in the proof of concept for the RDM infrastructures functionality validation.

In the future, further smart applications will be integrated into the infrastructure, such as scientific trial management, which provides two essential features for reliable and sustainable RDM. The first aspect of scientific trial management is the standardized creation of *Dataverses* and *Datasets* within the *Dataverse*. This is made possible via a web-based GUI in which using standardized catalogs *Dataverses* can be created or the metadata for projects *Dataset* can be specified. During the creation of a *Dataset*, the affiliation to a corresponding *Dataverse* can be established. The list of existing *Dataverses* is continuously updated to avoid duplicates. The use of standardized catalogs prevents different spellings and establishes a joint terminology among the institute’s researchers. This increases the findability of the data in the *Dataverse* via the metadata search. The second aspect of scientific trial management is the export of timer series data from the *InfluxDB* into a *Dataset* within the *Dataverse*. The export of time series data enables the movement of PT data to the *Dataverse* after a measurement series and thus increases its findability, accessibility, and reusability. An input mask is used to select the PT for which the data needs to be extracted and specify the Metadata on the basis of the standardized catalogs. The process gets triggered by an integrated button in the input mask. Based on the entered name the corresponding bucket is determined in the *InfluxDB* and the data for the specified period is retrieved. The microservice then creates a *Dataset* in the *Dataverse* according to the specifications and metadata of the input mask and saves the data in a structured, neutral tabular format to improve interoperability and reusability.

In addition to the scientific trial management efforts, prospective activities include the implementation of an application for the user-friendly creation of data management plans in the *Smart Application Space* in order to fully meet the requirements for a reliable RDM including the FAIR criteria to fully and sustainably document the project’s own data lifecycle. For this purpose, the established software RDMO will be integrated into the infrastructure, which will be connected to the existing microservices using its native API, offering the researchers a centralized and standardized tool for the creation of data management plans.

## 6. Proof of Concept and Evaluation

Following the implementation of the presented RDM architecture, a two-stage proof of concept with a respective concluding evaluation will demonstrate the advantages and the practicability of the authors’ architectural approach. First, a knowledge-based recommendation system is outlined to illustrate one use case and the benefits of a knowledge graph. Subsequently, the practical implementation of a DT is presented using the photometer. Afterward, it is demonstratively visualized by a real measurement series. Although the demonstration is based on a measurement series, the concluding evaluation is only qualitative. The focus of this work is the introduction of a novel infrastructure for RDM, not the investigation of a specific experimental context.

### 6.1. Knowledge-Based Recommendation of Measuring Devices

The first stage of the proof of concept is performed with a knowledge-based recommendation system to demonstrate the simplified identification of suitable measurement devices for generating research data enabled by the developed RDM infrastructure. For this purpose, three measuring devices have been selected and equipped with the necessary control system for their integration into the infrastructure and registration ability within *Eclipse Ditto*.

In addition to the photometer, the MIR scanner and the Multimodal Imaging System have been chosen because of their representative character. To enable this proof of concept, the individuals *OpticalMeasurementService*, *MFG_1* (corresponding to the photometer), *MFG_2* (corresponding to the Multimodal Imaging System), *MFG_3* (corresponding to the MIR scanner), *DiscreteDataPoint*, *HyperspectralImage*, *SkinLikeLiquid* and *MouseBrain* have been inserted into the ontology. The *OpticalMeasurementService* describes the type of offered service which is the same for all three devices. The individuals *MFG_1*, *MFG_2* and *MFG_3* represent the device’s manufacturer and the individuals *DiscreteDataPoint* and *HyperspectralImage* describe the generated data. The last two individuals *SkinLikeLiquid* and *MouseBrain* describe the target of the offered measurements, whereby they are only exemplary.

**Figure 7:**
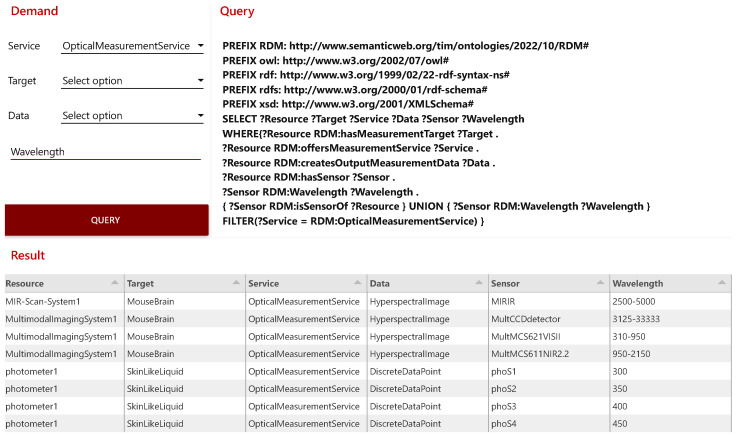
Node-RED Dashboard—Query Functionality for Specification-Driven Device Recommendation.

With the prepared ontology, the three devices are started, which triggers the process described in the implementation. After their boot, the devices register themselves in *Ditto* and are provided with a DT. Afterward, the DTs notify the DTOS of their registration, and the semantic representations are created automatically which can now be queried. Figure 7 shows the GUI. It is divided into the three areas *Demand*, *Query*, and *Result*. In the *Demand* area, various filters can be used to specify the demands on a measurement or a measuring device. The *Query* area displays the SPARQL query generated from the set filters by the microservice which is sent to the *Knowledge Graph*. The *Result* area lists the device recommendations based on the submitted query. The fewer filters are set, the more comprehensive the results are. Figure 7 therefore features all devices presented in the research landscape, as only the service is defined as an *OpticalMeasurementService*.

**Figure 8:**
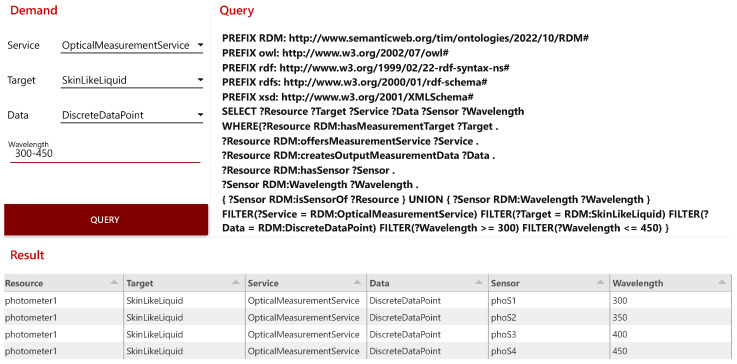
Node-RED Dashboard—Result for Detailed Specification-Driven Device Recommendation.

Subsequently, the *Node-RED* dashboard is used for a device recommendation based on the present use case. This use case requires an optical measurement service for a skin-like liquid to provide discrete data points as measured values. The liquid needs to be measured with a wavelength range of 300 to 450 nanometers. Figure 8 illustrates the result of the query with the specified filters. In the *Result* section, the recommended devices for the defined requirements are shown. For this use case, the photometer, which is able to cover the required wavelength range with its four sensors, provides an optical measurement service, and delivers discrete data points, is recommended.

### 6.2. Interfacing the Digital Twin of a Photometrical Measurement Device

In the second step, the continuous integration of the photometer and its DT is proven. In this case, *Node-RED*, which is located in the *Smart Application Space*, was utilized again to provide a GUI to the DT. This ensures an interface to the DT via the DTOS and *Eclipse Ditto* for parameterizing, operating, and monitoring the physical measurement experiment. The practical procedures of the demonstration experiment are described below.

**Figure 9:**
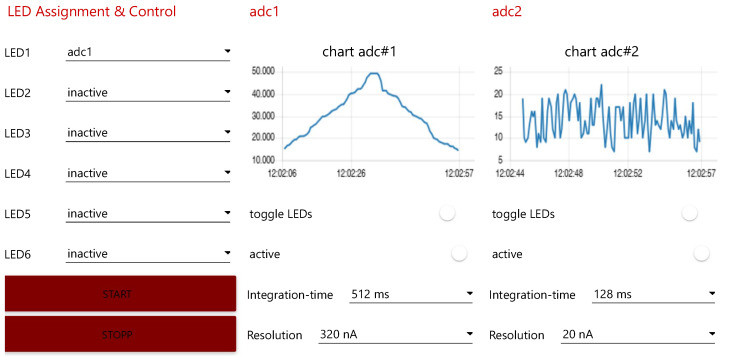
Node-RED Dashboard—Visualization and GUI of the Photometer Digital Twin Recording an Actual Measurement of a skin-like liquid Suspension (adc#1) and Reference Ambient Interference Measurement (adc#2).

A dilution series was performed on a skin-like liquid suspension with variable concentration of *New Coccine* (E124 Sigma Aldrich, St. Louis, MO, United States) (Figure 9). On the detector side, two EPD-660-1-0.9 [59] from Roithner Lasertechnik were used. An LED, type ELD-650-523 [60] from Roithner Lasertechnik, was utilized as the light source. The connection between the photometer and the probe is realized by optical fibers. *Adc1* measures the reflected light signal caused by different concentrations of *New Coccine*. *Adc2* measures the relevant ambient interfering light. The obtained digital values can be converted into a corresponding current value based on the set parameters. The resulting measurement signal, cleaned of interfering signals, can then be determined by subtracting these two values.

For a brief qualitative evaluation, the comparison with the previously applied procedure for data processing and storage can be referred to. Since the photometer did not have a network interface, data could only be read out via the serial interface of the controller. Refs. [41,42,43] employed manual methods to read out the measurement series, such as Matlab, Labview offer or directly writing down the data arriving via the serial interface within a text file. Afterward, they used tools like Excel or Matlab to evaluate the measurement results manually. As a consequence, the typical problems of data management arise again, i.e., many locally, decentrally distributed research data, missing access authorizations, and missing experimental references. The new concept of holistic engagement within the RDM infrastructure overcomes these problems and provides an integration platform. All data are labeled and referenced in the database with relevant experimental information and thus made accessible for future investigations. The parameterization of the photometer no longer has to be done by reprogramming the controller, but can now be conveniently adjusted via its GUI. This approach prevents maloperation, saves expert knowledge, workload, and, consequently, time. Henceforth, measurement apparatuses can be developed independently of use cases and used for experiments without any subsequent effort.

## 7. Discussion

This work elaborates an architecture for reliable as well as sustainable RDM tailored for the increasing amount of gathered data at a big interdisciplinary research institute. Facing the problems of multiple measuring sources, experimental devices, and the associated mass data that must be processed, the need for modern RDM arises. Guiding away from the conservative treatment of research data in a decentralized and passive archival manner, accompanied by issues in data loss, accessibility, interpretability, etc., a holonic infrastructure for managing devices, experiments, and their resulting data, entirely new opportunities arise to exploit the extensive potential of digitized RDM in research institutions. Addressing the obstacles of transposing RDM in a complete digitized form, the envisioned concept is leveraged by the DT paradigm. DTs act use case independently as a disruptive enabler technology in digital transformation. Therefore, the state of the art was examined and several requirements were identified in terms of RDM *(R-RDM1–R-RDM4)* and DTs *(R-DT1–R-DT6)*. Aligning the architecture to a sustainable modern RDM practice, the broadly accepted FAIR principles were utilized. Additionally, the derived requirements for DTs naturally fit well with the aforementioned FAIR principles and jointly build the foundation on the hereby outlined RDM infrastructure. Subsequently, all the requirements are set into context, followed by a brief overview of how they have been satisfied in the previous sections.

- In particular, *R-DT1: Replication, Representation and Interoperability* meets perfectly with *R-RDM1: Findable*, *R-RDM3: Interoperable* and *R-RDM4: Reusable*. Every PT is precisely described as DT within the *Digital Twin Space* facilitated by *Eclipse Ditto* and its JSON-based twin representation examined by the exemplary Photometer implementation. Herein embedded are all necessary structural and semantic information, which are further consumed by the DTOS which instantiates this information into *Apache Jena Fuseki’s* knowledge graph and thus establishing the pillar of later interaction and querying of all metadata within the *RDM Core Space*. To do so, the twins and their knowledge representation are unambiguously connected with each other.
- *R-DT2: Interconnectivity, Data Acquisition* fits well with *R-RDM2: Accessible*. The DTs settled within *Eclipse Ditto* are connected bidirectionally via various standard IoT interfaces (e.g., MQTT, HTTP, etc.) to their physical pendants. The different types of the DT’s data including its metadata are all covered by the dynamically updated JSON representation.
- *R-DT3: Data Storage* can be aligned with *R-RDM1: Findable*, *R-RDM2: Accessible*, *R-RDM3: Interoperable* and *R-RDM4: Reusable*. All types of data are managed, homogeneously stored, and labeled by the DTOS within both applied storage approaches. While *InfluxDB* is serving a time-series technique, the *Dataverse* offers a repository-based approach. The labeling relates to the metadata managed by the DTOS and the instantiated individuals within *Apache Jena Fuseki’s* knowledge graph. The data access can be achieved by calling DTOS API or in a two-staged manner by querying the knowledge graph and afterward pulling the data from the resulting storage locations.
- *R-DT4: Synchronization* could be satisfied in the demo implementation by using MQTT realized by *Eclipse Mosquitto* for the connection between the twins. Every change of state actualizes the DT and superordinated components or vice versa the PT.
- *R-DT5: Interface and Interaction* meets with *R-RDM2: Accessible* and *R-RDM3: Interoperable*. As well *Eclipse Ditto* as the entire *RDM Core Space* components offer open APIs to interact and request data. Even *Node RED* located in the *Smart Application Space* embodies basic GUI and interaction schemes of the DTs as a demonstrative implementation.
- *R-DT6: Optimization, Analytics, Simulation and Decision-Making* addresses several value-adding features on top of DTs in accordance with every FAIR principle. The *Smart Application Space* is intended to be the habitat of these value-adding features and applications which is founded on the subordinated three spaces. So far just *Node-RED* represents one demonstrative approach to highlight potential future functionalities. With the introduced RDM infrastructure in place, there are no restrictions and obstacles in the potential magnitude of later developable smart applications or tools.
- The last requirement *R-DT6: Security* is suitable regarding *R-RDM2: Accessible*. *Eclipse Ditto* supports state-of-the-art security standards including encryption, policy, and tenant-based DT management to gain proper access to required entities.

The contribution of the paper demonstrates that DTs are a perfectly suitable enabling technology for the central management of RDM entities and a reliable RDM itself. Further advantages can be drawn in the generation of knowledge and the reuse of data from other experiments or devices. Diversified datasets can be correlated with each other to gain entirely new insights. Especially for non-trivial human-readable data structures, i.e., multidimensional parameter arrays, this brings tremendous benefits. By processing research data in the way shown, inconsistencies, accessibility problems, data loss, etc., are no longer issues, also paving the way for more sustainability in research. Even the reusability of experimental knowledge can dissolve the need of reproducing difficult and energy-consuming experiment setups if still examined and well-labeled data are available. This also contributes to the minimization of the environmental footprint in research.

A self-developed photometer from the authors’ research institute is used as the first demo implementation of a PT and its DT. Subsequently, it is shown that the maximum depth of integration allows access to all the functionalities of the RDM infrastructure. Especially the reconfigurability of already manufactured physical devices through their DT offers great modification opportunities. i.e., measuring devices can be built use case agnostic and later parameterized by their DT as proven before.

The introduced knowledge graph is dedicated as the heart of the RDM infrastructure. Paving the way for intelligent interaction behavior between the DTs, it was initially proven that a measuring equipment recommendation can be established based on the DT’s knowledge representation.

However, the presented implementation covers mandatory sub-parts of the overall RDM infrastructure, the individual parts will need to be investigated at a much more fine-grained level in future proceedings. The authors are aware of the fact that this kind of infrastructure is only reasonable in large research institutions, where large amounts of diverse data accumulate. The optimum potential of such twinning architectures comes with a critical count of DTs. Improved scenarios for collaboration and interaction can then be explored. Another challenge arises from more complex measuring devices like non-customizable mass spectrometers. The ability of all research devices to communicate via the network and automatically aggregate data for the RDM involves an increased security risk and vulnerability to functional errors. Thus, future work has to cope with the analysis and integration of extremely heterogeneous research equipment into the RDM infrastructure. Furthermore, the development of experiment-specific representative metrics to ease the correlation between various previously examined results must be tackled. Additionally, the knowledge graph as a foundation for intelligent behavior must be further developed to realize a higher degree of action within the *Smart Application Space*. Henceforth, with a higher level of automation of the RDM infrastructure, matters of security, confidentiality, but also functional safety will be considered in the authors’ future work.

Summarizing with the overall rationales, the RDM infrastructure is intended to be introduced and used at the authors’ research institute within the upcoming 3 years empowered by a research project. All the researchers should be sensitized in terms of FAIR-compliant RDM. Thus, a better research domain independent cooperation between people should take place tackling problems together. To get them all on board and involved, it is absolutely necessary to pay the highest attention to usability and user-friendliness to avoid acceptance problems later on. This can be achieved by involving researchers from every domain in the development process of the RDM infrastructure.

## 8. Conclusions and Future Work

In order to cope with the increasing amount of generated data at research institutions, this paper introduced an infrastructure for handling research data produced by manifold heterogeneous measurement devices and experimental setups. Facing nowadays bigger growing requirements on RDM, the DT paradigm is utilized and highlighted as suitable enabler technology.

According to the analysis of relevant literature, the combination of both approaches results in a research gap, which this paper attempts to address. Requirements on reliable RDM, especially the FAIR principles, preserve value to researchers through the entire data lifecycle. In symbiosis with DT requirements, these principles could be afterward conceptually derived. Underlining the subsequent implementation, some of the typical measuring devices and apparatuses of the authors’ institute have been highlighted as well as the specific use case of a photometer which was implemented afterward. Built upon four hierarchical key pillars, the architecture splits from the bottom up in the *Physical Space*, the *Digital Twin Space*, the *RDM Core Space*, and the *Smart Application Space*. Through the example of the Photometer, a complete integration scenario was shown including every mandatory part of the RDM infrastructure. Further, a proof of concept showed the feasibility and advantages of the utilization of knowledge graphs as well as the beneficial functionalities of DTs. In the subsequent discussion, the individual requirements were put into context with each other along with the implemented architecture. The discussion revealed that DTs are the perfect companion for the realization of a reliable and sustainable RDM to gain added value.

As main contributions, this paper (1) introduced a novel approach for handling large amounts of research data according to the FAIR principles managing them in a centralized, structured manner; (2) obtained the necessity of utilizing DTs to overcome obstacles of the digital transformation within research institutes gearing them for Research 4.0; (3) developed a top-level knowledge graph addressing upcoming issues of interoperability and meta representation of experimental data and associated devices, paving the way for correlation of complex experimental data; (4) elaborated an implementation variant for reactive DTs as the base for later proactive realizations going beyond DTs as pure, passive state representations; (5) outlined a highly reconfigurable design approach shown by a photometer opening up entirely new possibilities with less development efforts in hard- and software engineering by utilizing the DT, not the physical device itself.

However, several limitations force future research rationales. To fully exploit the potential of the architecture, much research data needs to be collected, which is only feasible for large research institutes. A lot of work regarding the integration of measuring devices and experimental setups (e.g., non customizable mass spectrometers) needs to be done. Interaction schemes based on the knowledge graph must be elaborated to rise DTs to proactive behavior. Experiment-specific representative metrics must be envisioned to facilitate the correlation of the resulting data. In addition to that, smart applications have to be integrated into the *Smart Application Space* to gain the genuine added value of such infrastructures. This work covers mandatory sub-parts of the overall RDM infrastructure but the single parts have to be examined in a much more fine-grained manner in the authors’ future work. Likewise, issues of security, confidentiality, but also functional security will be considered forthcoming. In order to substantiate the practicability, future publications with experimental use case-specific data are planned.

## Figures and Tables

**Figure A1:**
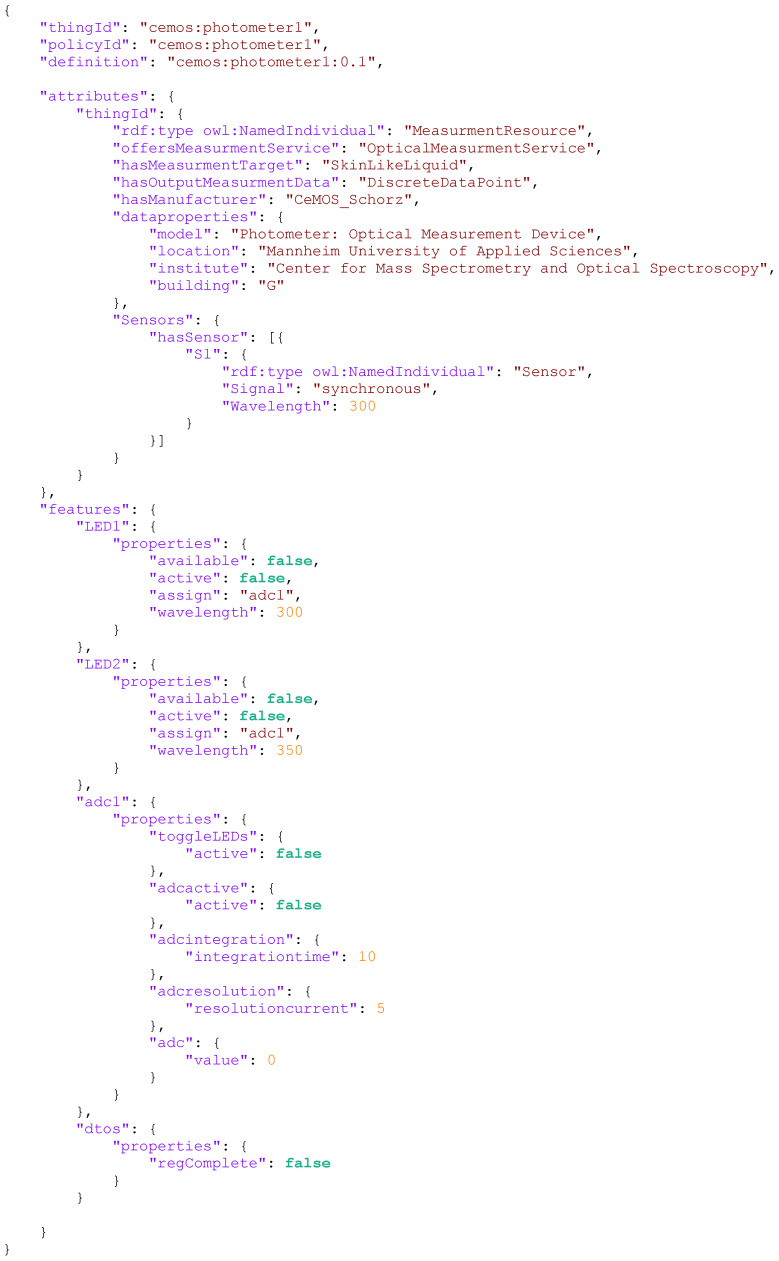
Eclipse Ditto Information Model.

## Data Availability

Not applicable.

## Appendix A

The JSON-formatted information model of *Eclipse Ditto* is shown on the next page. For reasons of clarity and space, *LED3–LED6*, as well as *adc2–adc4*, have been substituted. Their structure is analogous to the ones shown. Based on the physical structure of a PT, it is one of the main components of the DT representation. Thus, it serves not only as a data basis for all applications infrastructurally settled above it but also as a semantically enriched information model, which the DTOS uses to describe the device within the *Knowledge Graph*.
